# O02 Engaging health workers, students and the public to take action to tackle AMR: 10 years of the Antibiotic Guardian campaign

**DOI:** 10.1093/jacamr/dlae217.002

**Published:** 2025-01-23

**Authors:** D Ashiru-Oredope, M Onwunle, E Gilham, J Charlesworth

**Affiliations:** UK Health Security Agency, London, UK; UK Health Security Agency, London, UK; UK Health Security Agency, London, UK; UK Health Security Agency, London, UK

## Abstract

**Background:**

The Antibiotic Guardian campaign was developed in 2014 by Public Health England (now UK Health Security Agency, UKHSA). The initial concept for the website and logo was developed jointly with BSAC. The campaign was launched to increase engagement from healthcare professionals (HCPs) and the public to tackle antimicrobial resistance (AMR). It started as an online pledge system only; over the years the campaign has expanded to include Antibiotic Guardian schools outreach initiative, students AMR conference and shared learning events. Through collaborations with Africa CDC and WHO Europe the campaign also has pledges tailored for HCP and the public in Africa and translated pledges respectively. This study examined the campaign after 10 seasons (years) to assess engagement and describe the demographics of Antibiotic Guardians.

**Methods:**

Antibiotic Guardians’ demographics were collected when pledging online and used to produce descriptive statistics of pledge group, pledge chosen, geography, and how they heard about the campaign between 24 July 2014 and 31 December 2023. Data on organizations registering their antimicrobial stewardship (AMS) activities are collected via and downloaded from the Antibiotic Guardian website for analysis. Entries to the Antibiotic Guardian Shared Learning Awards and registrants to the Antibiotic Guardian Schools Ambassadors programme were collected via Google forms from 2016 and 2019 respectively to 2021, with Microsoft Forms subsequently used from 2022 to present. Questionnaire data was collated and analysed to provide total numbers of entries and registrants to the two programmes. Website visitors were described using Google analytics data.

**Results:**

From the inception of Antibiotic Guardian to the end of 2023 there were 852 713 unique website visitors resulting in a total of 177 681 pledges recorded via the main website English pledge page; 153 285 pledges from the UK, 9755 pledges from 199 other countries (6 continents with 78.5% from Europe) and 14 641 from unknown countries. Website visitors increased each year with peaks during European Antibiotic Awareness Day and World Antibiotic Awareness Week. The greatest increase in pledges and highest conversion rate (42%) occurred in 2020. A large number of pledges have been received from the public (12%, 21 249/177 681) and students (11%, 19 229/177 681). The most common method of learning about Antibiotic Guardian was through community pharmacy (29.6%, 52 620/177 681) with 4.1% (7316/177 681) hearing about the campaign through social media. Additionally, 995 organizations have registered their AMS activities through Antibiotic Guardian, 466 entries have been made to the Antibiotic Guardian Shared Learning and Awards events and the Antibiotic Guardian Schools Ambassadors programme has received over 400 registrants.

**Conclusions:**

The Antibiotic Guardian campaign continues to engage HCPs and the public to commit to tackling AMR, especially among those with prior awareness of the topic. The increase seen in pledges in 2020 was especially apparent in pharmacy teams and coincided with the introduction of a national Quality Scheme which required patient-facing pharmacy staff to become Antibiotic Guardians. This highlights the potential impact of policy on engagement with AMS initiatives. Future work needs to determine how engagement could be improved to increase pledges from HCPs outside of pharmacy teams.

